# Phenotypic and molecular analysis of nontypeable Group B streptococci: identification of *cps2a* and hybrid *cps2a/cps5* Group B streptococcal capsule gene clusters

**DOI:** 10.1038/s41426-018-0138-6

**Published:** 2018-08-08

**Authors:** Areej Alhhazmi, Gregory J. Tyrrell

**Affiliations:** 1 Division of Diagnostic and Applied Microbiology, University of Alberta, Edmonton, AB Canada; 2 Medical Laboratory Technology, College of Applied Medical Sciences, Taibah University, Madinah, Saudi Arabia; 3 The Provincial Laboratory for Public Health (Microbiology), Edmonton, AB Canada

## Abstract

The Group B streptococcus (GBS) can express a capsular polysaccharide (CPS). There are ten recognized CPSs (Ia, Ib, and II–IX). A GBS isolate is considered nontypeable (NT) when CPS cannot be identified as one of ten types. Two groups of GBS NT isolates were studied, isolates without surface sialic acid (sia(−)) and isolates with surface sialic acid (sia(+)). The first objective was to characterize NT sia(−) isolates that failed CPS identification by an immunodiffusion antisera typing assay and a RT-PCR capsule typing assay. NT sia(−) isolates were characterized by assaying phenotypic changes and identifying *covR/S* mutations that may potentially have a role in the altered phenotypes. The second objective was to characterize NT sia(+) isolates that failed to identify as one of the ten CPS types by an immundiffusion antisera-based typing assay and a RT-PCR capsule typing assay yet expressed capsule. Fifteen NT sia(−) isolates displayed increased β hemolysis/orange pigmentation, decreased CAMP activity, inability to form biofilm, and susceptibility to phagocytosis by human blood. DNA sequence analysis of the *covR/S* genes in the sia(−) isolates found mutations in 14 of 15 isolates assayed. These mutations in the *covR/S* genes may potentially contribute to lack of expression of phenotypic traits assayed in vitro. For the three NT sia(+) isolates, whole-genome sequence analyses identified two isolates with *cps* gene clusters identical to the recently described and uncommon CPSIIa type. The third isolate possessed a hybrid cluster containing *cps* genes for both CPSIIa and CPSV suggesting recombination between these two gene clusters.

## Introduction

Group B streptococci, GBS, (also referred as *Streptococcus agalactiae*) are a leading cause of invasive infections which can manifest as pneumonia, septicemia, and meningitis in neonates and a serious cause of morbidity and/or mortality in adults with underlying diseases^1–7^. GBS produce an array of virulence factors which include a β-hemolysin/cytolysin which can cause a narrow zone of β-hemolysis on 5% sheep blood agar, a CAMP factor that lyses sheep red blood cells previously sensitized with a sphingomyelinase produced by some *Staphylococcus aureus* strains^8,9^, and a capsular polysaccharide (CPS) of which there are ten types (Ia, Ib, and II–IX)^10–13^. These ten types of CPS can either be identified serologically or by various molecular assays. Those GBS that cannot be CPS typed are termed nontypable (NT). The NT phenotype of some GBS isolates may be due to the expression of undetectable amount of CPS by serological methods, lack of CPS expression, or production of uncharacterized capsular polysaccharide for which CPS typing antibodies not yet are available^14^.

Functions of CPS include protection of GBS from being killed by host immune cells such as macrophages and is a key component in the process of biofilm formation in the presence of human plasma^15^. Inactivation of CPS biosynthesis gene(s) reduces resistance toward phagocytic killing^16^ and inhibition of biofilm formation^15^.

GBS CPS of the ten CPS variants are formed by different arrangements of four component sugars (glucose, galactose, *N*-acetylglucosamine, and sialic acid) into a unique repeating unit^17–20^. All recognized GBS CPS types have sialic acid attached to their CPS structure^17–20^ that can interfere with complement-mediated killing by host immune cells^21,22^. Based on the conservation of sialic acid among all recognized GBS CPS types and the essentiality of its presence for full capsule biosynthesis and expression^17,18^, sialic acid has been utilized as recognition marker for GBS CPS production^23^.

Expression of GBS virulence factors, such as CPS, is controlled by the two-component system CovR/S. CovR/S is a major global regulatory system that is responsible for modulating the transcription of up to 7% of total GBS genomic genes^24^ including GBS virulence genes such β-h/c^25-29^, CAMP factor^25^, cell surface proteins^25^, capsule, and surface sialic acid expression^30^. Mutations in *covR/S* have been shown to alter the phenotypic expression of these virulence factors^24^.

We reported previously that 9% of GBS isolates in Alberta, Canada, collected from patients with invasive diseases between 2003 and 2013 were identified as NT^31^ and of those, 52.8% were sialic acid negative (sia(−))^23^. The sia(−) phenotype in NT GBS isolates suggests loss of *cps* expression as sialic acid is present on all known GBS CPS types. This suggested that the NT GBS isolates that were sia(−) in our collection may have genetic mutations in the *covR/S* genes resulting in a loss of CPS expression^25,27,28^. We also previously identified from this collection three sialic positive (sia(+)) GBS isolates that could not be CPS typed by a serological assay or by PCR^23^.

The first objective was to characterize NT sia(−) isolates that failed CPS identification by an immunodiffusion antisera-based typing assay and a RT-PCR capsule typing assay. The second objective was to characterize NT sia(+) isolates that failed to identify as one of the ten CPS types by an immundiffusion antisera-based typing assay and a RT-PCR capsule typing assay yet expressed capsule. This characterization included the identification of potential new *cps* gene clusters among these isolates using whole-genome sequencing.

## Results

### Sia(−) isolates

#### Phenotypic differences between GBS NT sia(−) isolates and GBS wild-type strain COHI

**Fig. 1:**
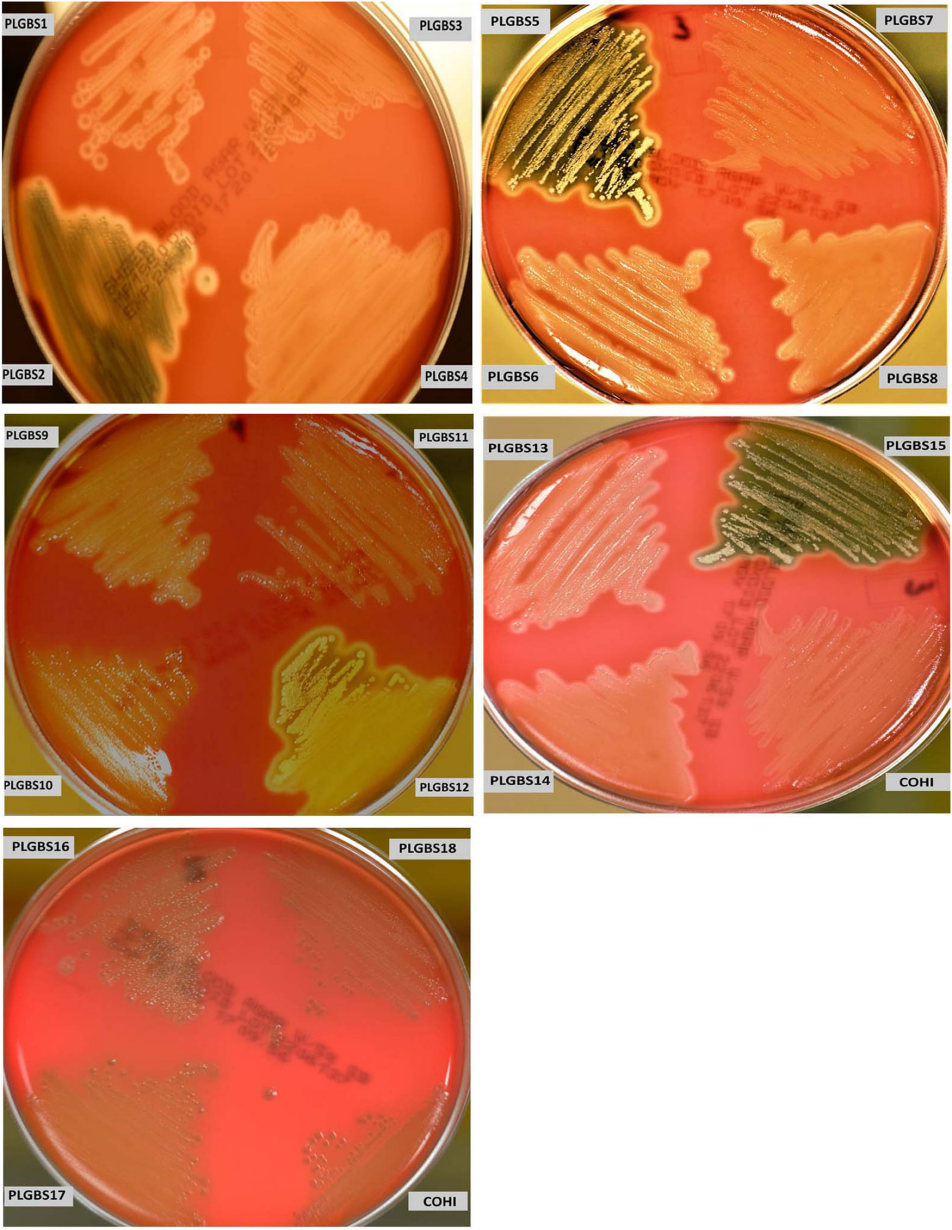
Hemolytic activity of sia(−) NT and sia(+) NT isolates on blood agar with 5% sheep blood. Bacteria were streaked onto a 5% sheep blood agar plate and incubated for 24 h at 37 °C. The GBS COHI isolate was included as control which displayed a narrow zone of hemolysis

Analysis of β-hemolytic activity in our collection of sia(−) isolates found that 15 sia(−) NT isolates (18%) exhibited increased β-hemolytic activity on 5% sheep blood agar compared to the positive control GBS isolate COH1 (Fig. 1). These were designated PLGBS1–15. The increase in the hemolytic activity on sheep blood agar plates was confirmed by a microtitre hemolysis assay, which showed an average fold increase of 3.5× (±0.8) (ranged from 2.6 to 4.36) over the control GBS strain COHI (Table 1). In addition to the increased hemolytic activity, these 15 sia(−) isolates visually displayed greater orange pigmentation vs. control (GBS strain COHI) as shown in culture pellets (supplemental Figure 1). This is expected as β-hemolysis and orange pigment production are caused by the same toxin, an ornithine rhamnolipid^29^. This data suggested that these sia(−) isolates may harbor mutations in their *covR/S* genes. Based on the enhanced β-hemolysis which suggested possible CovR/S changes, we focussed attention on only these 15 sia(−) NT GBS isolates in our collection of sia(−) isolates for subsequent analysis.

**Table 1:** The microtitre hemolysis assay for sia(−) NT isolates

Identifier	Hemolytic capacity^a^ % (±SD)	Fold change^b^
PLGBS1	81.5 (±0.1)	3.7
PLGBS2	58.0 (±0.08)	2.6
PLGBS3	85.5 (±0.02)	3.9
PLGBS4	78.0 (±0.2)	3.5
PLGBS5	96.1(±0.3)	4.4
PLGBS6	83.7 (±0.4)	3.8
PLGBS7	73.4 (±0.1)	3.3
PLGBS8	74.8 (±0.04)	3.4
PLGBS9	76.3 (±0.1)	3.5
PLGBS10	94.9 (±0.1)	4.3
PLGBS11	67.0 (±0.1)	3.0
PLGBS12	58.1 (±0.05)	2.6
PLGBS13	74 (±0.01)	3.4
PLGBS14	94.7 (±0.2)	4.3
PLGBS15	93.1 (±0.03)	4.2

^a^Hemolytic capacity: the hemolytic capacity of a given strain was determined by dividing the absorbance of a given isolate by the positive control (0.1% SDS) after subtraction of the negative control (PBS)^27^

^b^Fold change measured by dividing the hemolytic capacity of an isolate by the positive control (COHI)

To confirm that the enhanced hemolytic activity and increase in orange pigmentation are reflected at the expression level in those isolates, we assayed *cylE* transcription. *cylE* (a gene encoded in *cyl* operon) is essential for GBS β-hemolytic production/orange pigmentation. RT-PCR using primers specific for the *cylE* gene revealed increases in *cylE* mRNA production in all sia(−) isolates assayed (average fold increase of 4.3 × ±2.3) (Supplemental Table 1). This suggested the enhanced β-hemolysis/orange pigment was due to increases in *cyl* operon transcription.

Assays for CAMP factor activity showed that these 15 isolates displayed no reaction or weaker CAMP activity on 5% sheep blood in comparison to the control isolate (COHI) (Supplemental Figure 2). Also, RT-PCR with primers specific for *cfb*, the gene responsible for the CAMP factor phenotype^32^, showed a reduction in *cfb* mRNA transcript in comparison to the control (Supplemental Table 1).

In addition to β-hemolytic activity/orange pigmentation production, and CAMP activity, we assayed the growth rates of the 15 sia(−) isolates in comparison to the GBS control isolate COHI in TH (an enriched medium) and RPMI (a minimal defined medium). In TH broth, the 15 sia(−) isolates displayed a faster growth rate compared to the control strain, with an average division time of 57 min (±0.01) compared to 64 min for COH1. In RPMI, all 15 sia(−) isolates with increased β-hemolytic activity were unable to grow in RPMI unlike the control which reached an average OD of 0.5 (±0.06) after 16 h of incubation.

To further demonstrate that the 15 sia(−) isolates did not produce polysaccharide capsule, we assayed for *cpsE* gene transcription. *cpsE* is a gene encoding a glycosyltransferase that initiates the biosynthesis of polysaccharide repeating units contained in the capsule^33^. Loss of *cpsE* gene expression can lead to lack of CPS expression. It was found that *cpsE* transcription was downregulated to very low levels in all sia(−) isolates in comparison to the COH1 control (Supplemental Table 1). This suggested the decrease in expression of the *cpsE* gene has led to loss of capsule expression in these GBS isolates.

#### Reduction in biofilm formation of the 15 sia(−) isolates

**Fig. 2:**
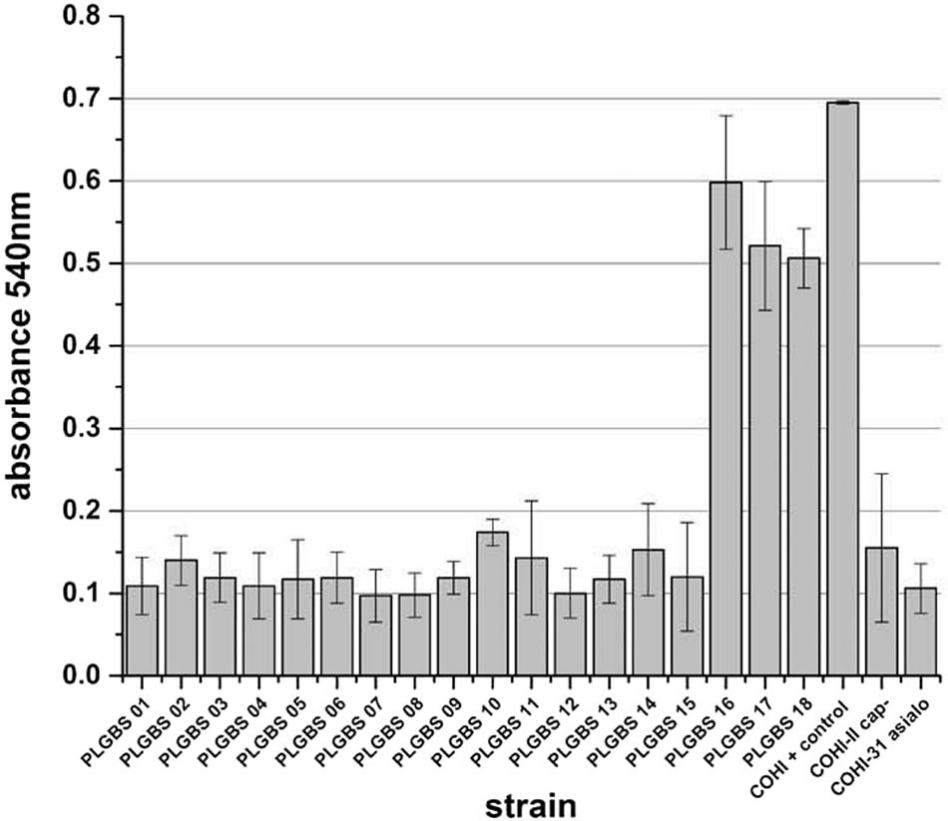
Biofilm formation of sia(−) NT and sia(+) NT isolate. Bacteria were grown at in RPMI supplemented with 1% glucose and 20% human plasma (HP). Cells were stained with crystal violet. Quantification was performed by solubilization of the stained biomass in ethanol/acetone (80/20) and measuring the absorbance at 540 nm. COHI was included as positive control while unencaspulated (COHI-II) and asialo (COHI-31-15) mutants were negative controls

Previous research has shown that GBS CPS plays a major role in biofilm formation, which is important for GBS persistence and pathogenicity^15^. We hypothesized that the sia(−) isolates in our collection would display a reduced ability to form biofilm. The unencapsulated and asialo mutants (negative controls) were impaired in their ability to form biofilm with an average absorbance at 600 nm of 0.2 (±0.09) and 0.1 (±0.03), respectively (Fig. 2). The positive control, COHI, was able to form biofilm with an average absorbance at 600 nm of 0.695 (±0.002). GBS isolates that were sia(−) were unable to form biofilm having an average absorbance of 0.1 (±0.02, *p* > 0.001) (Fig. 2).

#### Loss of resistance toward phagocytic killing by the 15 sia (−) isolates

**Fig. 3:**
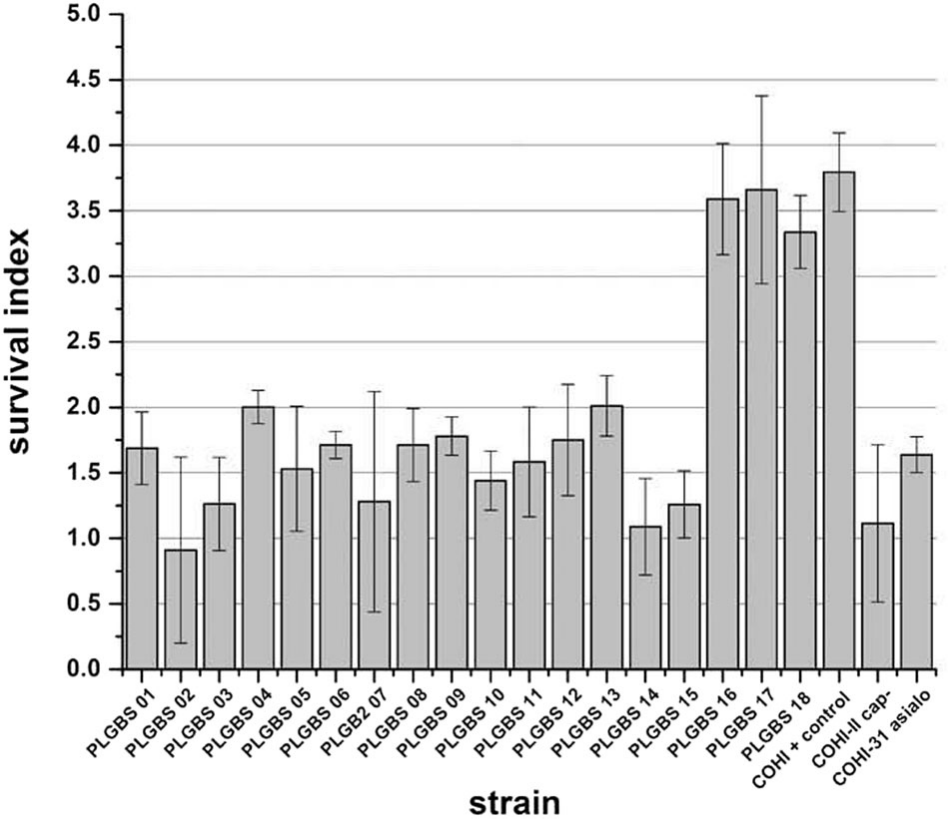
Human whole-blood killing assay of sia(−) NT and sia(+) NT isolates. Bacteria (10^3^ CFU/100 µl) were mixed with 300 µl of freshly drawn human blood in heparinised tubes, and incubated for 3 h with agitation at 37 °C, and dilutions were plated on blood agar for enumeration of CFU. Survival index was calculated as follows: (CFU at the end of the assay)/ (CFU at *t* = 0 h). GBS COHI was included as negative control while unencapsualted (COHI-II) and asialo (COHI-31-15) mutants were included as positive controls

It has previously demonstrated that GBS CPS inhibits complement deposition, thereby reducing oposonophagocytotic clearance and contributing to immune resistance^22^. In addition, GBS isolates that display low hemolytic activity and high CPS expression have an increased resistance toward host immune defense. In contrast, isolates that exhibited high hemolytic activity and low capsule expression were phagocytosed in an oposonophagocytotic assay^25,27^. Based on these observations, we hypothesized that sia(−) isolates may be more susceptible to host immune defenses. To determine this, the survival of these GBS isolates in a human whole-blood killing assay was assessed^27,30^. The 15 sia(−) isolates displayed a low survival index with an average value of 1.5 (±0.4, *p* > 0.001) (ranged from 0.8 to 2) compared to the positive control (survival index value of 3.8 (±0.3)). The negative controls, the unencapsulated and asialo GBS mutants, had low survival indices of 1.1 (±0.6) and 1.6 (±0.1), respectively (Fig. 3).

#### Genetic analysis of the *covR/covS* genes of sia(−) NT isolates with increased β-hemolytic activity and orange pigment

Enhanced β-hemolytic activity, increased orange pigment production, loss of CAMP activity, inability to grow in minimal essential media, and loss of capsule expression strongly suggested mutations in the *covR/S* two-component regulatory system had occurred in these 15 sia(−) isolates^25–29^. To verify whether the changes in the phenotypes of the 15 sia(−) isolates in this study was due to mutations that could potentially alter the amino acid coding sequence of CovR or CovS, the *covR* and *covS* genes were sequenced.

DNA sequencing of *covR* and *covS* genes identified mutations in the *covR* and *covS* sequences leading to the predicted amino acid changes shown in Table 2 for 14 of the sia(−) isolates. PLGBS1 did not display any nucleotide changes in *covR* and *covS* genes. Eight of the 15 sia(−) isolates displayed mutations that predicated protein truncations of CovR (PLGBS2, 5, 8, 10, 11, 12, 13, and 15). Only three sia(−) isolates showed DNA sequence mutations in *covS* (PLGBS4, 12, and 14). Also, the DNA sequence of *covS* for PLGBS14 predicted an amino acid substitution of 21 amino acids in CovS. The remaining DNA sequence mutations predicted two–four amino acid substitutions in CovR (PLGBS3, 6, 7, and 9) (Table 2). As PLGBS1 did not display any changes in the nucleotide sequences of *covR* and *covS*, it is likely other genes are involved in lack of CPS expression.

**Table 2:** The predicted amino acid changes in CovR and CovS based on nucleotide mutation(s) in the *covR* and *covS* genes identified among 15 sia(−) NT GBS isolates

Isolate designation	Predicted amino acid changes in CovR/S
PLGBS1	No *covR* or *covS* mutations detected therefore no changes
PLGBS2	CovR: truncation at amino acid 2
PLGBS3	CovR: Leu20Phe, Glu21Gly
PLGBS4	CovS: Val1Ile, Met185Thr, Leu225Phe
PLGBS5	CovR: truncation at amino acid 2
PLGBS6	CovR: Asp210Glu, Ile215Leu
PLGBS7	CovR: Ile209Met, Ile211Leu
PLGBS8	CovR: truncation at amino acid 2
PLGBS9	CovR: Leu19Ser, Leu20Val
PLGBS10	CovR: truncation at amino acid 2
PLGBS11	CovR: truncation at amino acid 2
PLGBS12	CovR: truncation at amino acid 2 CovS: Val333Met
PLGBS13	CovR: Ile4Asp, Ile6Asn, Ile7Asn, Glu8Gly, Asp9Arg and truncation at amino acid 10
PLGBS14	CovS: substitution of amino acids 253 to 274 with Asp-Val-Ala-Val-Val-Lys-Gly-His-Ile-Gly-Leu-Leu-Gln-Arg-Trp-Gly-Lys-Asp-Asp-Pro-Asp
PLGBS15	CovR: truncation at amino acid 2

### Sia(+) isolates

Knowing that the presence of surface sialic acid can predict the presence of polysaccharide capsule, it was unusual to find in our collection three GBS isolates (PLGBS16, PLGBS17, and PLGBS18) which failed to react with antisera raised against the nine known CPS types (Ia, Ib, and II–VIII) yet were sialic acid positive^23^ (data not shown). Additionally, these three isolates could not be genotyped by our previously described RT-PCR typing assay (which includes Ia, Ib, and II–IX)^23^, suggesting novel mechanisms of encapsulation. A polysaccharide stain revealed that these three GBS isolates microscopically displayed CPS surrounding the cell wall (Supplemental Figure 3). These observations prompted us to further analyze these three GBS isolates with unidentifiable polysaccharide capsules.

#### Phenotypic properties of GBS sia(+) NT GBS isolates

PLGBS16, PLGBS17, and PLGBS18 isolates exhibited β-hemolytic activity similar to the wild-type control, COHI (Fig. 1), grew as a white colony (Supplemental Figure 1), and displayed enhanced CAMP activity (Supplemental Figure 2). Growth rate comparisons in TH (enriched media) and RPMI (minimal media) of the three sia(+) NT GBS isolates compared to the control GBS COHI isolate showed that they have similar growth rates with division times of 66 (PLGBS16), 65 (PLGBS17), and 63 (PLGBS18) minutes compared to 64 min for COHI. The growth profile for the three isolates in RPMI were similar to the control with an average OD of 0.55 (±0.12), 0.59 (±0.09), and 0.6 (±0.06), respectively, after 16 h of incubation.

#### Biofilm formation by sia(+) NT GBS isolates

Based on the important role of GBS capsule in biofilm formation^15^, we hypothesized that the three sia(+) GBS isolates were able to form biofilm unlike the 15 previously characterized sia(−) isolates. All three isolates were able to form a biofilm in RPMI media containing 20% human plasma and 1% glucose^15,34–36^ (average absorbance at 600 nm of 0.5 (±0.05)) similar to the positive control COHI (0.7, ±0.002) (Fig. 2). Negative controls (the unencapsulated and asialo mutants) were impaired in their ability to form biofilms with an average absorbance at 600 nm of 0.2 (±0.09) and 0.1 (±0.03), respectively (Fig. 2).

#### Resistance of phagocytic killing by sia(+) NT GBS isolates

To assay resistance to phagocytic killing, the ability of the three sia(+) NT GBS isolates to survive in fresh human blood was assayed in a human whole-blood killing assay^27,30^. The three isolates displayed high-survival indices with an average index of 3.5 (±0.2) similar to the positive control COHI strain (survival index 3.8, ±0.3) (Fig. 3), indicating that three sia(+) isolates were able to resist killing likely due to the presence of the capsule.

#### Genetic analysis of sia(+) NT GBS isolates

As PLGBS16, PLGBS17, and PLGBS18 represented potentially new GBS CPS variant(s), whole-genome sequence analyses were done focusing on the MLST and *cps* gene comparative analysis. MLST data for PLGBS16, PLGBS17, and PLGBS18 were assigned a ST1 designation for PLGBS16 and PLGBS17 that were included in clonal complex (CC) 1. ST1/CC1 (http://pubmlst.org/sagalactiae/) has been reported to be commonly found among CPSV isolates^37^. PLGBS18 was assigned a ST2/CC1 designation.

**Fig. 4:**
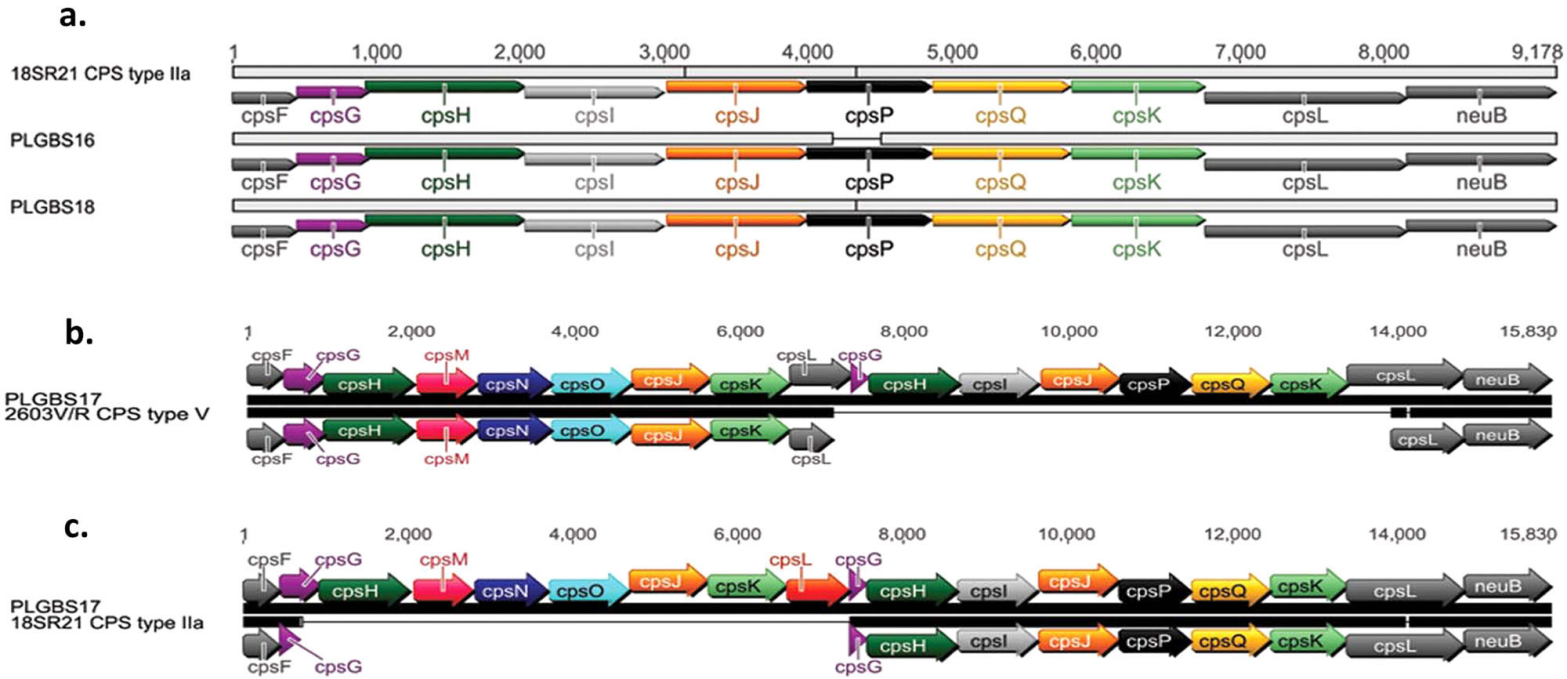
Comparative analyses between cps-specific sequences. **a** 18SR21 (CPSIIa) vs. PLGBS16 and PLGBS18, **b** PLGBS17 vs. 2603V/R (CPSV), and **c** PLGBS17 vs. 18SR21 (CPSIIa)

To investigate whether isolates PLGBS16, PLGBS17, and PLGBS18 encode novel genes involved in CPS synthesis, we compared the DNA sequences of the *cps* gene clusters of these isolates to the *cps* gene cluster of known CPS types^33^. Comparative analysis of the *cps* gene cluster revealed that PLGBS16 and PLGBS18 and the previously sequenced *cps2a* gene cluster (18SR21, GenBank accession #AAJO01000077) shared highly homologous sequences across the *cps* locus (Fig. 4 and Table 3). Poyart et al. have previously suggested that CPSII is encoded by two *cps* clusters, with suggested designation subtypes IIa and IIb represented by 18SR21 and AY375362 cps sequences^38^. PLGBS16 and PLGBS18 shared low homology with *cps* sequences of CPSIIb (GenBank accession #AY375362) (Table 3). A CPS dot blot assay using earlier prepared in-house rabbit antibody raised against CPSII did not identify these isolates as CPSII, suggesting that the in-house antibody preparation targeted CPSIIb only. In contrast to PLGBS16 and PLGBS18, the isolate-designated PLGBS17 isolate shared highly homologous sequences with CPSIIa (18SR21) and CPSV (2603V/R) (Fig. 4 and Table 3)^38,39^. Two well-conserved regions spanning from *cpsA* to *cpsF* and from *neuB* to *neuA*, flanking a central region between *cpsG* and *cpsL*, were found in PLGBS17 (Fig. 4). In the central region, the PLGBS17 isolate showed a high similarity to CPS-determining region of CPS type V, including the following genes *cpsG*, *cpsH*, *cpsM*, *cpsN*, *cpsO*, and *cpsL*. It also shared high similarity to *cps2a*-specific genes including a fragment of *cpsG* (228 bp), and *cpsH*, *cpsI*, *cpsQ*, *cpsS*, and a fragment of *cpsL* (725 bp) (Fig. 4 and Table 3). These results suggest that cps-specific region of PLGBS17 is a hybrid of CPSIIa and V. Further analysis using in-house-prepared rabbit polyclonal antibodies raised against CPSII and commercial antibodies against CPSV failed to react in a CPS dot blot assay (data not shown). Together, the data suggested a novel hybrid CPS type composed of *cps2a* and *cps5* genes.

**Table 3:** Nucleotide sequence identity comparisons for individual cps genes for the 3 sia(+) PLGBS16, PLGBS17, and PLGBS18 vs the control type strains (18SR21 CPSIIa, AY375362 CPSIIb and AE009948 CPSV)

18SR21 Type IIa	*cpsG*%	c*psH*%	*cpsI*%	*cpsJ*%	*cpsP*%	*cpsQ*%	*cpsK*%	*cpsL*%
PLGBS16	100	100	100	99.9	61.5	100	100	100
PLGBS17	100	100	100	99.9	100	100	100	98.2
PLGBS18	100	100	100	99.9	99.9	100	100	100
**AY375362** **Type IIb**	***cpsG*** **%**	***cpsH*** **%**	***cpsI*** **%**	***cpsJ*** **%**	***cpsP*** **%**	***cpsQ*** **%**	***cpsK*** **%**	***cpsL*** **%**
PLGBS16	73.7	47.2	56.5	49.7	Absent	Absent	86.4	99.9
PLGBS17	30.3	47.2	56.5	49.7	Absent	Absent	86.4	98.1
PLGBS18	73.7	47.2	56.5	49.7	Absent	Absent	86.4	99.9
**AE009948** **Type V**	***cpsG*** **%**	***cpsH*** **%**	***cpsJ*** **%**	***cpsM*** **%**	***cpsN*** **%**	***cpsO*** **%**	***cpsK*** **%**	***cpsL*** **%**
PLGBS17	100	100	100	100	100	100	100	58.2

## Discussion

The polysaccharide capsule is an important virulence factor of GBS and a convenient target for typing to aid in the understanding of GBS epidemiology. GBS strains for which a polysaccharide type cannot be determined by serological assays are usually reported as a nontypable as they typically do not express a capsule. The use of molecular assays targeting *cps* genes in GBS have aided in identifying the *cps* genes in these nontypable GBS isolates thereby assigning a GBS type even though no capsule may be expressed. In addition to lack of capsule expression, a subset of GBS that are nontypable may actually express a capsule that is unrecognizable using serological or molecular assays. Identifying and understanding the prevalence of those GBS strains that do express CPS yet are considered NT can provide information regarding disease trends of these unencapsulated yet invasive bacteria.

To better understand the NT phenotype of GBS isolates, we undertook a detailed characterization of a collection of nontypable GBS collected from cases of invasive disease in Alberta. For the sia(−) isolates, we focussed on potential changes in GBS that were likely caused by changes in the two-component GBS global regulatory system CovR/S. As CovR/S controls the expression (or lack of) of an array of phenotypes, we focussed only on those isolates that were hyperhemolytic/hyperpigmented and were CAMP factor negative. These phenotypic attributes plus loss of biofilm formation and susceptibility to phagocytic killing strongly suggested changes had occurred in the CovR/S system. DNA sequencing of both the *covR* and *covS* genes identified an array of mutations that had occurred in these genes.

A number of *covR/S* mutations were discovered in 14 out of the 15 hyperhemolytic NT sia(−) isolates. It is interesting that half of the *covR/S* mutations (7/14), potentially resulted in truncations of CovR at amino acid number 2. This suggests that there was a loss of almost the entire CovR protein in these GBS isolates and serves as a logical explanation for loss of capsule production. As this mutation occurred frequently in our collection, it is possible this represents a non-encapsulated clone circulating in the Alberta population. However, a close examination of the years in which these isolates were collected from cases of iGBS disease shows a wide-temporal period reducing the likelihood of the isolates being clonal.

With respect to CovS, three isolates were found to have DNA sequence mutations in the *covS* coding sequence potentially resulting in CovS amino acid changes. One change in particular was the substitution of a 21 amino-acid-long stretch of amino acids located at the C-terminal end of CovS (PLGBS14). *covR* or *covS* mutations are not unusual as a variety of mutations in these genes have been previously reported. Whidbey et al. previously reported a hyperhemolytic/hyperpigmented GBS with a Glu120Pro substitution in CovR^40^. Also, work by Almeida et al. reported a collection of mutations in the *covS* gene. These were primarily single amino acid substitutions (Thr43Ile, Ala87Val, Gly133Ser, His209Asn, Trp297Leu, and Glu337Ser) and the one double substitution (Trp297Cys and Gly298Trp)^41^. None of these amino acid changes were predicted amino acid changes in the *covR* and *covS* mutations found in our isolates suggesting mutations of *covR* and *covS* may occur in multiple locations in these genes. Understanding the frequency of *covR/S* mutants as they occur is important as this two-component regulatory system coordinates a number of virulence factors including vaccine targets. It is unclear if *covR/S* mutants would be favored if a GBS vaccine is introduced that contains one or more virulence factors that can be downregulated by mutations in *covR/S*, such as CPS.

The finding of three GBS sia(+) isolates with non-serological and non-PCR typable *cps* gene clusters was unexpected as it has been established that there are only ten known CPS variants designated Ia, Ib, and II–IX. However, based on this study and the work of others, it is clear that a second *cps2* gene cluster exists for GBS isolates from cases of invasive disease in humans^38^. The small number of reports for CPSIIa in the literature suggests that CPSIIa is relatively rare in occurrence possibly explaining why it has not been classed as one of the well-established CPS types. Differences between the two *cps2* gene clusters is evident in the literature; however, a clear recognition that two CPSII capsules exist is generally lacking. Berti et al. have previously compiled a nucleotide sequence identity table for all ten CPS types listing each *cps* gene^19^. For CPSII, these were reported as *cpsA*, *cpsB*, *cpsC*, *cpsD*, *cpsE*, *cpsF*, *cpsG*, *cpsH*, *cpsI*, *cpsJ*, and *cpsK*. Recently, Kapatai et al. compared the cps loci of all ten serotypes^42^. In this report, the *CPSII* genes were designated *cpsA*, *cpsB*, *cpsC*, *cpsD*, *cpsE*, *cpsF*, *cpsG*, *cpsH*, *cpsI*, *cpsJ*, *cpsP*, *cpsQ*, and *cpsK*. The major difference between the two gene clusters being the presence of *cpsP* and *cpsQ* in one report and absent in the other. It is highly likely CPSIIb (no *cpsP* and no *cpsQ*) is the more common CPSII type and is probably the CPSII serotyped by most laboratories performing GBS serotyping. To be accurate, GBS CPS typing reports should split CPSII types into IIa and IIb even though CPSIIa is likely uncommon. This would extend the GBS CPS typing collection to 11 types (Ia, Ib, IIa, IIb, III, IV, V, VI, VII, VIII, and IX). The genes *cpsP* and *cpsQ* from the *cps2a* gene cluster could be easily incorporated into PCR-based CPS typing schemes allowing investigators to readily identify CPSIIa types.

It was interesting that even though CPSIIa is uncommon, a *cps2a/cps5* hybrid gene cluster was discovered in our nontypable collection. This *cps2a/cps5* hybrid (genes *cpsH*, *cpsI*, *cpsQ*, *cpsS*, *cpsK*, and a fragment of *cpsL* from *cps2a*+ genes *cpsG*, *cpsH*, *cpsN*, *cpsM*, *cpsO*, *cpsK* from *cps5*) failed to react with either CPSII antisera (now known to be CPSIIb antisera) or CPSV antisera from our antisera collection. The finding of only one isolate with this hybrid *cps* gene sequence suggests that this is a rare event and likely does not give much selective advantage over other cps types, otherwise more isolates with this CPS type would have likely been found.

In summary, characterization of a collection of NT sia(−) isolates found these bacteria did not express a variety of phenotypic traits previously shown to be regulated by *covR/S*. Also, characterization of the NT sia(+) isolates found two isolates that contained a *cps* gene cluster designated *cps2a* and an isolate that contained a novel hybrid *cps* gene cluster comprised of genes from the *cps2a* and *cps5* gene clusters.

## Materials and methods

### Bacterial strains, growth conditions, and oligonucleotides

The relevant characteristics of the bacterial strains used in this study are listed in Table 4. We reported previously that 159/1683 GBS isolates collected from patients between 2003 and 2013 were identified as NT^31^ and of those, 84 of the 159 were sialic acid negative (sia(−))^23^, whereas 75/159 were sialic acid positive (sia(+))^23^. Of the 75 serologically nontypable sia(+) isolates, only three isolates (PLGBS16, PLGBS17, and PLGBS18) could not be genotyped by our previously described RT-PCR typing assay (which included Ia, Ib, and II–IX)^23^. All GBS isolates were cultured in TH broth or Columbia blood agar plates (Dalynn Biologicals, Calgary, Canada) containing 5% sheep blood or RPMI 1640 (Thermo Fisher Scientific, Toronto, Canada) as a synthetic medium. GBS liquid cultures were grown in standing-filled flasks. All incubations were at 37 °C.

**Table 4:** Strains used in the study

Strain designation	Genotype or phenotype	Reference
COHI	CPS type III	50
COHI-II	Emr, Tcr, Tn9J6AE, asialo, CPS type III	18
COHI-31-15	Tcr, RfU, Smr, Tn916 cap−, CPS type III	51
NCS6	CPS type II	52
NCS13	CPS type V	52
PLGBS1-PLGBS15	Sia(−) and NT	23
PLGBS16, PLGBS17, PLGBS18	Sia(+) and NT	23

*Emr* erythromycin resistant, *Tcr* tetracycline resistant, *Rfr* rifampin resistant, *Smr* streptomycin resistant, *asialo* sialic acid negative, *cap−* capsule negative

### Assay for hemolytic activity

GBS hemolytic activity was assayed as previously described^27^. Briefly, an overnight culture (10^9^ cfu) in TH broth were centrifuged for 5 min at 3000 × *g*, washed twice with phosphate-buffered saline (PBS), and re-suspended in 1 ml of PBS. In a 96-well conical-bottom microtiter plate (MP Biomedicals, Santa Ana, USA), 100 µl per well (10^8^ cfu) of the bacterial resuspension was placed in the first well, and serial twofold dilutions in PBS were performed across the plate, each in a final volume of 100 µl. An equal volume of 1% sheep erythrocytes washed once with PBS (5 min by centrifugation at 3000 × *g* to avoid non-specific red blood cell lysis), and re-suspended in PBS, was then added to each well, and the plate was incubated at 37 °C for 60 min.

PBS alone and 0.1% SDS were used as negative and positive controls for hemolysis, respectively. After incubation, the plates were centrifuged at 3000 × *g* for 10 min to pellet the unlysed red blood cells and GBS, and 100 µl of the supernatant was transferred to a replica plate. Hemoglobin release was assessed by measuring A_420_, and the hemolytic capacity of a given strain was determined. Briefly, the positive control absorbance value (0.1% SDS) and absorbance values of each isolate were subtracted from the negative control value (PBS) (normalized values). The normalized values of each isolate were divided by the normalized positive control absorbance value and multiplied by 100. This value was designated as the hemolytic capacity.

All assays were performed in triplicate, repeated three times, and the mean value ± standard deviation (SD) is indicated.

### Assay for orange pigment production

The broth cultures were incubated to the stationary phase of growth (18 h) and then centrifuged. The pellet was spotted onto nitrocellulose membrane (20 µl/spot) using a bio-dot apparatus (Bio-Rad, Hercules, USA), which was dried for 30 min at room temperature.

### RNA isolation

THB broth (20 ml) was inoculated (1:20) with an overnight culture of GBS strains and incubated at 37 °C. Exponentially growing cells (OD_600_ 0.3–0.4) were harvested for 2 min at 6000 × *g* at 4 °C. The pellets were re-suspended by vortexing in 400 ml of resuspension buffer (12.5 mM Tris, 5 mM EDTA and 10% glucose). The supernatant was transferred to a fresh tube, and 1 ml of Trizol reagent (Thermo Fisher Scientific, Toronto, Canada) was added. The sample was incubated for 5 min at room temperature. Total RNA was extracted twice with chloroform-isoamyl alcohol (24:1, v/v) and precipitated in 0.7 volumes of isopropanol. After a washing step with 70% ethanol, the RNA pellet was dissolved in sterile DNase and RNase-free water (Thermo Fisher Scientific, Toronto, Canada) and treated for 30 min at 37 °C with RNase-free DNase I (1 unit per mg of total RNA) in 50 mM Tris-HCl (pH 7.5) and 10 mM MnCl_2_. DNase was inactivated by phenol–chloroform extraction, and the RNA was precipitated and washed with 70% ethanol, re-dissolved in RNase-free water and quantified by absorbance at 260 and 280 nm. Purity and integrity of RNA were controlled on agarose gels, and RNA was stored at −20 °C until use.

### Detection of mRNA by RT-PCR

Using a high capacity cDNA reverse transcription kit with RNAse inhibitor (Thermo Fisher Scientific, Toronto, Canada), 0.8 µg RNA was reverse transcribed as recommend by manufacture. RT-PCRs were carried out by using Fast SYBR® Green Master Mix (Thermo Fisher Scientific, Toronto, Canada). Reactions were carried out in a final volume of 20 ml containing 0.5 µg of RNAs, 0.5 μM of each forward and reverse primer (Integrated DNA Technology, IDT, USA) for each *cpsE*, *cylE*, *cfb*, and *rpsL* and 10 µl of master mix. The real-time PCR was performed using a TaqMan RT-PCR Applied Biosystems System 7500 (Applied Biosystems, Culver City, USA). The cycling conditions were denaturation at 95 °C for 1 min, followed by 40 cycles of amplification at 95 °C for 3 s, 60 °C for 30 s. The rate of temperature increase was 1 °C/s (or 0.5 °C/s), and fluorescence was acquired once. Each RT-PCR was repeated three times. The list of oligonucleotides used in this study is indicated in Supplementary Table 1. The differences between the genes *cylE*, *cpsE*, and *cfb* (genes assayed) vs. the housekeeping gene (*rpsL*) in NT sia(−) isolates and the same genes in the control COHI strain were calculated. These values were subtracted from each other (test – control, (ΔCTE − ΔCTC)) to determine the ddCT value. Fold change was calculated based on log2-ddCT. The average and standard deviation of the fold change were calculated from three independent experiments.

### Genomic DNA extraction

Genomic DNA extraction was performed as follows. Overnight broth cultures (1.5 ml) were centrifuged for 10 min at 3000 × *g*. Genomic DNA was re-suspended in 500 µl of 1× PBS (8 g NaCl, 0.2 g KCl, 1.44 g Na_2_HPO_4_, 0.24 g KH_2_PO_4_, and 1000 ml H_2_O [pH 7.2]) and washed two times with PBS. The pellet was used to extract genomic DNA using the Qiagen DNA mericon kit (Qiagen, Dusseldorf, Germany). Extracted genomic DNA was concentrated and dissolved in 30 µl Qiagen elution buffer or water and stored at −20 °C. RNase pretreatment was done prior to quantification of genomic DNA.

### PCR amplification and sequencing of *covR* and *covS*

PCR assays that target either *covR* or *covS* based on the sequences from GBS strain 2603V/R (Genbank accession #AE009948) were developed (Supplemental Table 2). The amplified fragments were sequenced using the same primers, covR-F and covR-R or covSA-F and covSA-R and covSB-F and covSB-R (Integrated DNA Technology, Coralville, USA) (Supplementary Table 1) used for amplification and compared to the GBS genome (Genbank accession #AE009948) available in Genbank.

### Biofilm forming assay

The biofilm forming assay was performed as described previously^15^. Briefly, GBS were grown in TH (Becton Dickinson, Franklin Lakes, USA) broth. Human plasma was collected from a human volunteer. Overnight GBS cultures were used to inoculate RPMI-glucose or RPMI-glucose supplemented with human plasma at an OD_600_ of 0.1, vortexed briefly and 180 µl volumes dispensed in 96 wells plate (MP Biomedicals, USA) and incubated for 18 h at 37 °C. The OD_600_ of each culture was measured to ensure that all cells had reached stationary phase with a similar density, and the cells were washed twice in PBS and air-dried for 15 min. Biofilms were stained with 0.4% crystal violet for 30 min and the wells were washed twice with PBS and air-dried. The bacterial biomass was then re-suspended for quantification in ethanol/acetone (80/20) solution and OD_540_ was measured. When OD values were above 1, twofold dilutions were performed for accuracy. The assay was performed in triplicate and at least three independent experiments were performed.

### Human whole-blood killing assay

Human whole-blood killing assay was performed as described previously^27,30^. Briefly, GBS was grown to early logarithmic phase, washed, and re-suspended in PBS. Inocula of 10^3^ CFU in 100 μl were mixed with 300 μl of freshly drawn human blood in heparinised tubes, and incubated for 3 h with agitation at 37 °C, and dilutions were plated on blood agar for enumeration of CFU. The survival index was calculated as follows: (CFU at the end of the assay)/(CFU at *t* = 0 h). The assay was performed in triplicate and repeated two times independently.

### Whole-genome sequencing

PLGBS16, PLGBS17, and PLGBS18 isolates were sequenced using a MiSeq instrument with an average sequencing depth of 120× (Illumina, San Diego, USA). Library preparation was performed using a tagmentation process. One nanogram genomic DNA as input and the Illumina Nextera XT Library preparation kit (FC-131-1096) as per manufacturer’s protocol (Illumina, San Diego, USA) was used. Resultant libraries were purified from free primers using Ampure beads (1:0.8 reaction mix:bead ratio). Libraries were qualified using the Agilent Bioanalyzer (Agilent, Santa Clara, USA) and quantified using a Qubit fluorimeter and Qubit HS DNA reagents (Thermofisher Scientific, Toronto, Canada). Libraries were loaded at 10 pM on an Illumina MiSeq v2 reagent kit (MS-102-2003) and processed at 2 × 500 cycles with 1% PhiX control DNA (FC-110-3001). Sequence data for the three genomes have been deposited in the NCBI genome database (BioProject PRJNA433769). Illumina reads were assembled to contigs by CLC genomic bench work using de novo assembly. The *cps* operons of PLGBS16 and PLGBS18 were assembled in one contig, whereas the *cps* locus of PLGBS17 was built from five different contigs encoding *cps* genes. The assembled genome was annotated using online annotation websites Rapid Annotation of Prokaryotes using Subsystem (RAST)^43^. Contigs corresponding to the chromosomal region encompassing the *cps* operon were identified and aligned by Muscles using Geneious and by using as query sequence the sequence of strain 18SR21 (GenBank accession ##AAJO01000077)^32^ or 2603V/R (GenBank accession# AE009948)^33^ cps region.

### Multilocus sequence typing (MLST) assay and assignment to clonal clusters

Multilocus sequence typing (MLST) was carried out as described previously^44^. Briefly, the seven housekeeping genes (*adhP*, *pheS*, *atr*, *glnA*, *sdhA*, *glcK*, and *tkt*) were assigned an allele number based on their sequences for the isolates sequenced. Each isolate was assigned a sequence type (ST) based on the allelic profile of the seven amplicons for each strain were grouped into CCs using the eBURST software program^45,46^. eBURST was set at the default setting that identified groups of related STs using the most stringent (conservative) definition. All members assigned to the same group shared identical alleles at six of the seven loci with at least one other member of the group (http://pubmlst.org/sagalactiae/).

### Double immunodiffusion assay for GBS CPS typing

CPS typing was performed using the Lancefield heat-acid extraction followed by a double immunodiffusion method as described previously^47,48^ for sia(+) isolates, PLGBS16, PLGBS17, and PLGBS18. The immunodiffusion assay of GBS CPS typing used for this study was based on reactions with antisera raised against cps types Ia, Ib, II, III, IV, V, VI, VII, and VIII. The type-specific antisera panel was prepared in rabbits within the laboratory^47,48^.

### CPS dot blot assay

A dot blot assay to detect CPS was performed as previously described with minor modifications^49^ for the sial(+) NT GBS isolates, PLGBS16, PLGBS17, and PLGB18. Late exponentially growing bacteria were washed in phosphate-buffered saline (PBS) and re-suspended in PBS to give an optical density at 600 nm of ~2 (Beckman Coulter, Mississauga, Canada). The bacterial suspension was spotted onto nitrocellulose membrane (20 µl/spot) using a bio-dot apparatus (Bio-Rad, USA), which was dried for 30 min at room temperature. The membranes were washed for 15 min with TBS (6.05 g Tris, 8.76 g NaCl in 1000 ml of H_2_O [pH 7.5]), incubated with blocking buffer (5% skim milk, 0.1% Tween 20 in TBS) for 60 min at 37 °C. The membrane was subsequently washed with TBS for 15 min. CPS was detected using specific rabbit polyclonal antibodies raised against CPSII (in-house preparation), CPSIII, or CPSV (Statens Serum Institute, Copenhagen, Denmark) at 1:1000 dilution. The secondary horseradish peroxidase-coupled anti-rabbit secondary antibody (Promega, Madison, USA) was used at 1:50,000 dilution. The membrane was washed 3 × 15 min with TBST and 2 × 15 min with TBS before developing with SigmaFast BCIP/NBT (Sigma-Aldrich, St. Louis, USA) for ~5 min. Development was stopped using three changes of distilled water.

## Electronic supplementary material

Supplemental figure legends and tables

Supplemental figure 1

Supplemental Figure 2

Supplemental Figure 3
