# Supplementary material for: Phenotypic and molecular analysis of nontypeable Group B streptococci: identification of cps2a and hybrid cps2a/cps5 Group B streptococcal capsule gene clusters
Source: Emerg Microbes Infect. 2018 Aug 8;7:137. doi: 10.1038/s41426-018-0138-6 (PMC6081472; doi:10.1038/s41426-018-0138-6)
Supplement: Supplementary file 1 — Supplemental figure legends and tables [file 41426_2018_138_MOESM1_ESM.docx]

**Supplemental**

Supplemental Figure 1. Orange pigmentation of sia(-) NT and sia(+) NT isolates.

In TH broth, a rich medium, late stationary phase cultures of sia(-)NT isolates were spotted on nitrocellulose membrane using bio-dot apparatus. GBS COHI was a used as a control as well as sia (+) NT isolates (PLGBS16, PLGBS17, and PLGBS18).

Supplemental Figure 2. CAMP activity for sia(-) NT and sia(+) NT isolates.

Bacteria were streaked on 5% sheep blood agar plates and incubated for 24 h at 37°C. The size of the area of complete lysis of red blood cells between *S. aureus* and GBS isolates reflected the level of CAMP expression. There was very faint lysis between *S. aureus* and sia(-) NT isolates as compared with the control. Isolates (PLGBS16, PLGBS17, and PLGBS18) were sia(+) NT isolates and displayed CAMP activity similar to the control.

Supplemental Figure 3. Capsules visualized using Anthony’s capsular polysaccharide stain.

PLGBS17 was grown in milk broth for 18 h to provide a proteinaceous background for contrast. The smear was stained with 1% crystal violet for 2 minutes and then rinsed gently with a 20% solution of copper sulfate. PLGBS17 is only shown. PLGBS16 and PLGBS18 displayed similar smears.

Supplemental Table 1. Expression of the messenger RNA of *cylE*, *cfb*, and *cpsE* genes for GBS sia(-) NT isolates

| **GBS designation** | ***cylE***  **fold change^a^ (±SD)** | ***cpsE***  **fold change^a^ (±SD)** | ***cfb***  **fold change^a^ (±SD)** |
| --- | --- | --- | --- |
| PLGBS1 | 2 (±0.4) | 0.04 (±0.03) | 0.6 (±0.7) |
| PLGBS2 | 2.1 (±0.8) | 0.12 (±0.05) | 0.1 (±0.04) |
| PLGBS3 | 6.4 (±1.3) | 0.42 (±0.5) | 0.1 (±0.02) |
| PLGBS4 | 2.2 (±0.8) | 0.03 (±0.03) | 0.1 (±0.1) |
| PLGBS5 | 8 (±1) | 0.03 (±0.01) | 0.1 (±0) |
| PLGBS6 | 4.6 (±0.3) | 0.01 (±0) | 0.1 (±0) |
| PLGBS7 | 3.1 (±1.2) | 0.01 (±0) | 0.03 (±0) |
| PLGBS8 | 2.2 (±0.5) | 0 (±0) | 0.1 (±0.2) |
| PLGBS9 | 2.5 (±0.3) | 0.04(±0.01) | 0.1 (±0.04) |
| PLGBS10 | 5.1 (±3.7) | 0.03 (±0.03) | 0.7 (±0.03) |
| PLGBS11 | 2.1 (±0.2) | 0.04 (±0.04) | 0.1 (±0.1) |
| PLGBS12 | 7.4 (±1.9) | 0.03 (±0.01) | 0.2 (±0.24) |
| PLGBS13 | 2.4 (±0.2) | 0.04 (±0.02) | 0.1 (±0) |
| PLGBS14 | 7.4 (±0.1) | 0.02 (±0.1) | 0.03 (±0.01) |
| PLGBS15 | 6.2 (±1.8) | 0.04 (±0.02) | 0.1 (±0.01) |
| COHI | 1 | 1 | 1 |

^a^ The differences between the assayed genes and the housekeeping genes (*rpsL*) in sia(-) NT isolates and the genes assayed and the *rpsL* in the COHI were calculated. These are ΔCT values for the tested genes (ΔCTE) and the control (ΔCTC) conditions, respectively. Then, the difference between ΔCTE and ΔCTC (ΔCTE-ΔCTC) was calculated to arrive at the Double Delta (dd) CT Value (ddCT). Fold change was calculated based on log2-ddCT. The average and standard deviation of the fold change were calculated from three independent experiments.

Supplemental Table 2: Primers used in study

| primers | sequences | reference |
| --- | --- | --- |
| cpsE-F | GTTTGCTCATATGTGGCATTGT | This study |
| cpsE-R | ATCCTACCATTACGACCTACTCT | This study |
| cfb-F | TTTCACCAGCTGTATTAGAAGTA | 32 |
| cfb-R | GTTCCCTGAACATTATCTTTGAT | 32 |
| cylE-F | CTGAAGCTTCCTTAGAAG | 53 |
| cylE-R | TGCCATTTGGAGAGATAAG | 53 |
| rpsL-F | GGACGTTTAGCACCGTATTTAGAAC | 53 |
| rpsL-R | CCTAAAAAACCTAACTCTGCCCTTC | 53 |
| covR-F | CGA TAA TTT TTT TGA CAGAG | This study |
| covR-R | TTA AGT CTC GCT ATC GAA C | This study |
| covSA-F | TTATATTTCTTTAGTTTCTTCAAA | This study |
| covSA-R | CAC GAC TTA ATG CAT CAG AT | This study |
| covSB-F | TGTCGACGAGTATAGTCTT | This study |
| covSB-R | GTG AAA AAT AAA AAA GAT CAG | This study |
