# Supplementary figures and images for: Phenotypic and molecular analysis of nontypeable Group B streptococci: identification of cps2a and hybrid cps2a/cps5 Group B streptococcal capsule gene clusters
Source: Emerg Microbes Infect. 2018 Aug 8;7:137. doi: 10.1038/s41426-018-0138-6 (PMC6081472; doi:10.1038/s41426-018-0138-6)

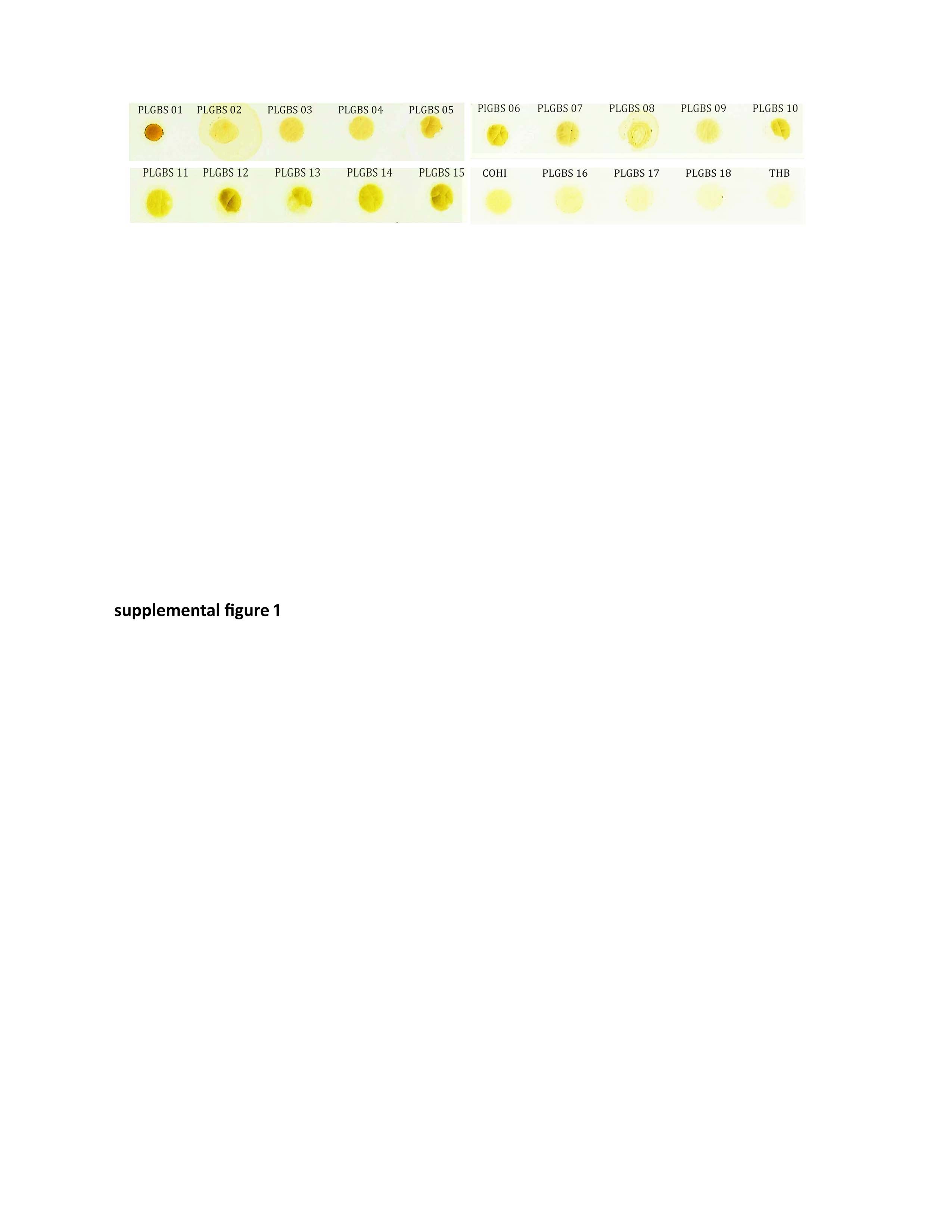

Supplement: Supplementary file 2 — Supplemental figure 1 [file 41426_2018_138_MOESM2_ESM.jpg]

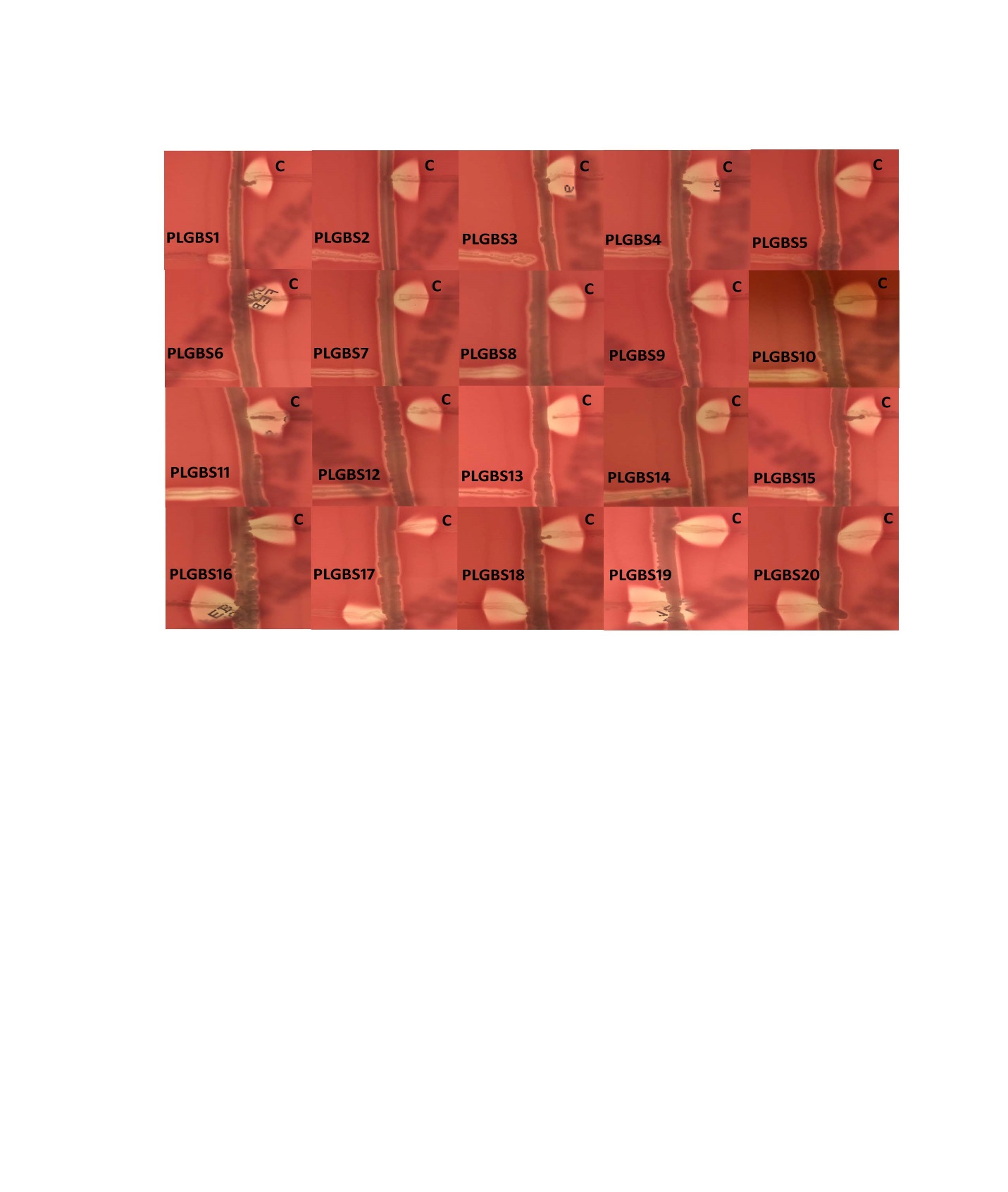


**Supplemental Figure 2.**

Supplement: Supplementary file 3 — Supplemental Figure 2 [file 41426_2018_138_MOESM3_ESM.docx]

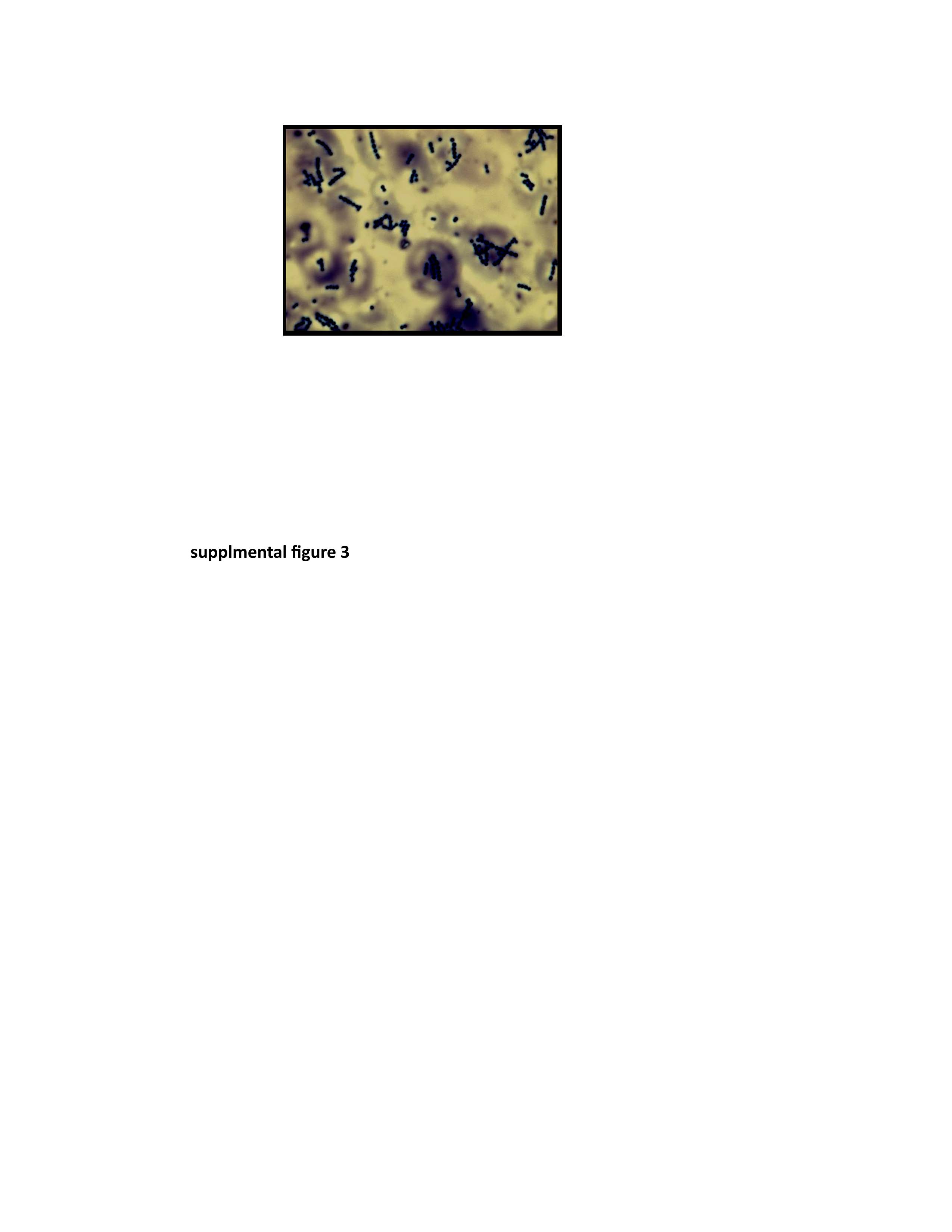

Supplement: Supplementary file 4 — Supplemental Figure 3 [file 41426_2018_138_MOESM4_ESM.jpg]
